# Genetic heterogeneity of primary lesion and metastasis in small intestine neuroendocrine tumors

**DOI:** 10.1038/s41598-018-22115-0

**Published:** 2018-02-28

**Authors:** Dirk Walter, Patrick N. Harter, Florian Battke, Ria Winkelmann, Markus Schneider, Katharina Holzer, Christine Koch, Jörg Bojunga, Stefan Zeuzem, Martin Leo Hansmann, Jan Peveling-Oberhag, Oliver Waidmann

**Affiliations:** 1 Department of Internal Medicine I, Johann Wolfgang Goethe-University Hospital, Theodor-Stern-Kai 7, 60590 Frankfurt, Germany; 2 Dr. Senckenberg Institute of Pathology, Johann Wolfgang Goethe-University Hospital, Theodor-Stern-Kai 7, 60590 Frankfurt, Germany; 3 Neurological Institute (Edinger-Institute), Johann Wolfgang Goethe-University Hospital, Heinrich-Hoffmann Str. 7, 60528 Frankfurt, Germany; 4 German Cancer Research Center (DKFZ), 69120 Heidelberg, German Cancer Consortium (DKTK), Partner Site Frankfurt/Mainz, 60590 Frankfurt, Germany; 5 CeGaT GmbH, Paul-Ehrlich-Straße 23, 72076 Tuebingen, Germany; 6 Department of General and Visceral Surgery, Johann Wolfgang Goethe-University Hospital, Theodor-Stern-Kai 7, 60590 Frankfurt, Germany; 7 Department of General surgery, Section for Endocrine Surgery, University Hospital Gießen Marburg, Baldinger Straße, 35043 Marburg, Germany; 8 Department for Gastroenterology, Hepatology and Endocrinology, Robert-Bosch-Hospital, Auerbachstraße 110, 70376 Stuttgart, Germany; 9 University Cancer Center Frankfurt (UCT), Johann Wolfgang Goethe-University Hospital, Theodor-Stern-Kai 7, 60590 Frankfurt, Germany

## Abstract

Data on intratumoral heterogeneity of small intestine neuroendocrine tumors (SI-NETs) and related liver metastasis are limited. The aim of this study was to characterize genetic heterogeneity of 5 patients with SI-NETs. Therefore, formalin-fixed, paraffin-embedded tissue samples of primary and metastatic lesions as well as benign liver of five patients with synchronously metastasized, well differentiated SI-NETs were analyzed with whole exome sequencing. For one patient, chip based 850k whole DNA methylome analysis was performed of primary and metastatic tumor tissue as well as control tissue. Thereby, 156 single nucleotide variants (SNVs) in 150 genes were identified and amount of mutations per sample ranged from 9–34 (mean 22). The degree of common (0–94%) and private mutations per sample was strongly varying (6–100%). In all patients, copy number variations (CNV) were found and the degree of intratumoral heterogeneity of CNVs corresponded to SNV analysis. DNA methylation analysis of a patient without common SNVs revealed a large overlap of common methylated CpG sites. In conclusion, SI-NET primary and metastatic lesions show a highly varying degree of intratumoral heterogeneity. Driver events might not be detectable with exome analysis only, and further comprehensive studies including whole genome and epigenetic analyses are warranted.

## Introduction

Neuroendocrine tumors of the small intestine (SI-NET) represent the most common small intestine neoplasm, occurring with an incidence of 1/100,000^1^. They originate from enterochromaffine cells of the digestive tract and frequently secrete neuroamines or peptide hormones which can lead to a variety of clinical syndromes. SI-NETs are usually well differentiated and most often show a low proliferation rate as well as a high percentage of distant metastases at diagnosis and 5-year survival rates are less than 50% in patients with metastatic disease^2,3^.

The only curative treatment of SI-NETs is complete surgical resection. In the majority of advanced tumors, treatment usually involves surgery of the primary lesion and mesenteric metastases to reduce symptoms caused by carcinoid syndrome, bowel obstruction or mesenteric ischemia and to prevent future complications. In addition, treatment with somatostatin analogues reduces hormone-related morbidity and prolongs time to progression^4,5^. Apart from Everolimus for treatment of nonfunctional neuroendocrine tumors, no standard second-line systemic treatment is currently available^6,7^. Treatment options include cytotoxic therapy, angiogenesis inhibitors, and radiolabeled somatostatin analogues^8^. For example, a recent phase III trial demonstrated that lutetium-177-Dotatate leads to a longer progression-free survival compared to high-dose octreotide LAR in patients with disease progression during first-line somatotstatin analogue therapy^9^. Development of targeted therapy approaches is impeded by only limited available data on genetic alterations and lack of potential driver genes of SI-NETs.

To date, three studies investigated the genomic landscape of SI-NETs with large scale next generation sequencing^10–12^. Thereby, SI-NETs were found to be genetically silent neoplasms with a mutational load of less than 1 mutation per mega base (Mb). Compared to the majority of other malignancies where mutation rates vary between 1 and >10/Mb, SI-NET can be regarded as one of the most silent neoplasms^13^. Data on variation of the mutational landscape between primary and metastatic lesions are limited and available exome data are highly diverse with the percentage of common mutations of primary lesion and metastasis ranging from 0–47%^11^. Further characterization of this intratumoral heterogeneity is of high importance to better understand tumorigenesis of SI-NETs and its possible implications on future therapy approaches.

In the present study we characterized the mutational landscape of pairs of primary tumor and hepatic metastases of well differentiated, synchronously metastasized SI-NETs from five patients. In addition, we performed DNA-methylome analysis in a patient with marked heterogeneity. We thereby unveiled a profound genetic variability strongly suggesting presence of subclonality within SI-NETs.

## Results

### Patients

Five patients with a SI-NET and synchronously diagnosed hepatic metastases were included. All tumors were positive for Synaptophysin, Chromogranin A, and CD56 and well/moderately differentiated (G1/G2). Ki-67 was between <2% and 15. Estimated mean percentage of tumor cells was comparable for primary tumors (mean 80%, range 70–90%) and metastatic lesions (86%, 70–95%). One patient had a resection in curative intention but experienced recurrent disease after one year. At study closure, one patient had controlled tumor burden under therapy with somatostatin, in the other four patients 2^nd^ and 3^rd^ line therapies were applied due to progressive disease. Mean follow-up was 46 mo (25–65 mo) and 2/5 patients had passed away at study closure. A summary of clinical data is provided in Table 1.

**Table 1:** Patient characteristics. Clinical characteristics of the study cohort. P: primary, M: metastasis, hep: hepatic, os: osseous, M.r.s.: Musculus rectus superior, SST: somatostatin analogue, TACE: transarterial chemoembolization, PRRT: Peptide Receptor Radionuclide Therapy, Tem: temozolomide, Cap: capecitabine, Ox: oxaliplatin, Bev: Bevacizumab, SD: stable disease.

PAT ID	SEX	AGE	G	KI67 P	KI67 M	T	N	M	L	V	PN	R	THERAPY	STATUS
1	F	60	G1	<2%	<2%	3	1	hep.	1	0	1	0	SST -> SD	alive
2	M	57	G2	10%	10%	3	0	hep., os.	1	1	1	1	SST/TACE/PRRT -> SD	alive
3	F	67	G2	10–15%	10–15%	4	1	hep.	1	1	1	0	SST ->SST/TACE ->PRRT -> TACE/SST	alive
4	M	68	G2	4–5%	<2%	3	0	hep.	1	1	1	0	SST, Everolimus, Tem/Cap, Cap/Ox/Bev	deceased
5	M	52	G1	<2%	5%	2	1	hep., os., M.r.s.	1	1	0	0	SST	deceased

### Mutational analysis

**Figure 1:**
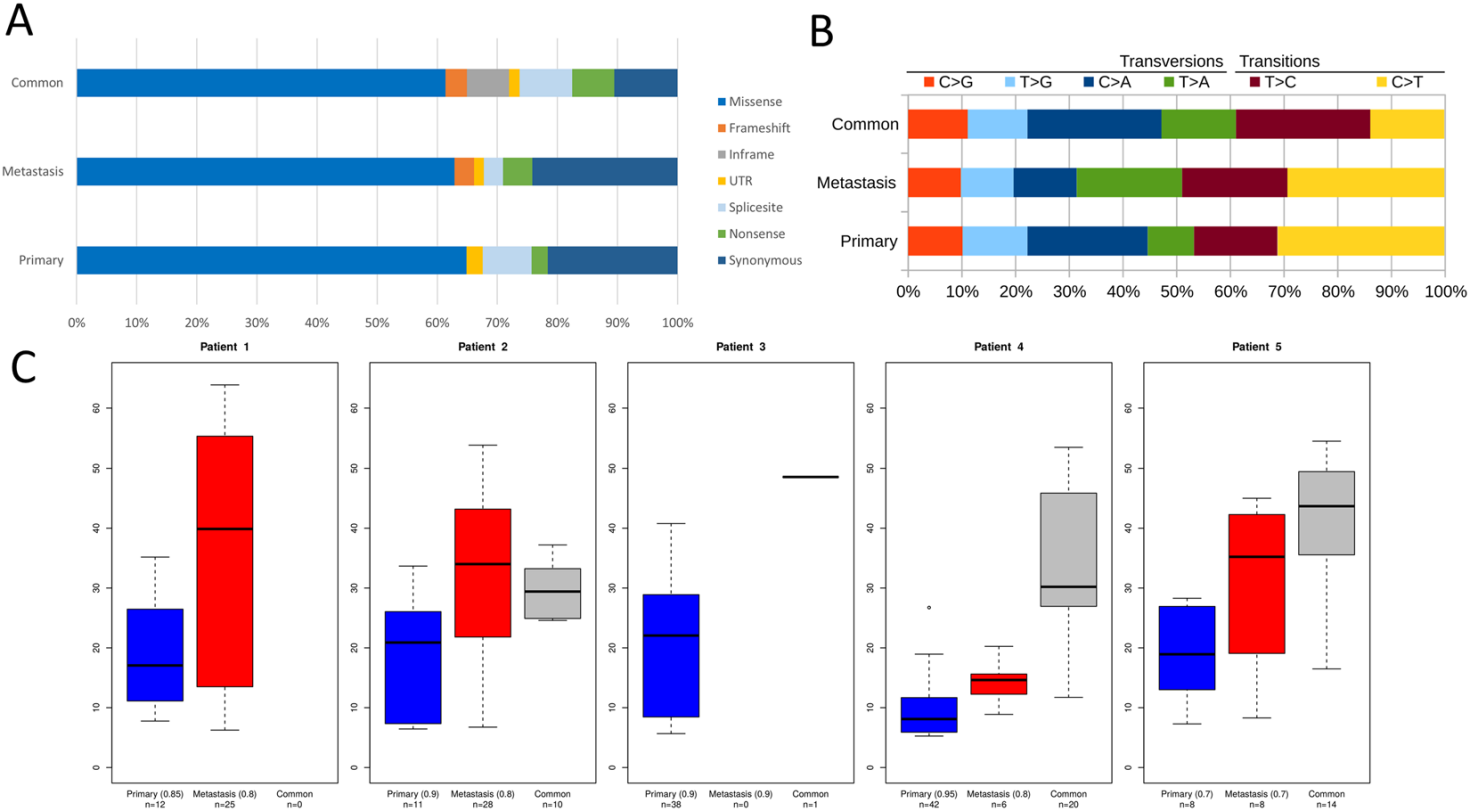
Mutational characteristics. Mutational details of exome sequencing of primary and metastatic samples of Patient 1–5. (**A**) Mutational categories according to mutations found only in the metastasis sample, only in the primary sample or in both samples (common). (**B**) Distribution of transversions and transitions according to presence in primary, metastasis or both (common). (**C**) Distribution of allele frequencies (AF) of mutations detected only in the primary lesion, only in the metastasis, or in both samples (common). Observed allele frequencies were scaled to account for estimated tumor content (given in brackets).

Exomes of all 15 tumor and non-tumor samples were successfully sequenced. A mean of 102 Mio reads (range 73–124 Mio) per sample was mapped. After removal of duplicates (mean 39%, range 31–54%), mean coverage depth was 120 reads per sample (range 87–146, Supplementary Table 2). Overall, 156 mutations in 150 genes were identified. Mean mutation rate per megabase was 0.36, while 0.31 mutations per megabase were identified in primary lesions and 0.40 in metastases, respectively, and amount of mutations per sample ranged from 9–34 (mean 22). 25 mutations were successfully validated with pyro- or Sanger sequencing (validation rate 100%). Functional categories and mutational characteristics are shown in Fig. 1A/B.

### Somatic intratumoral heterogeneity

**Figure 2:**
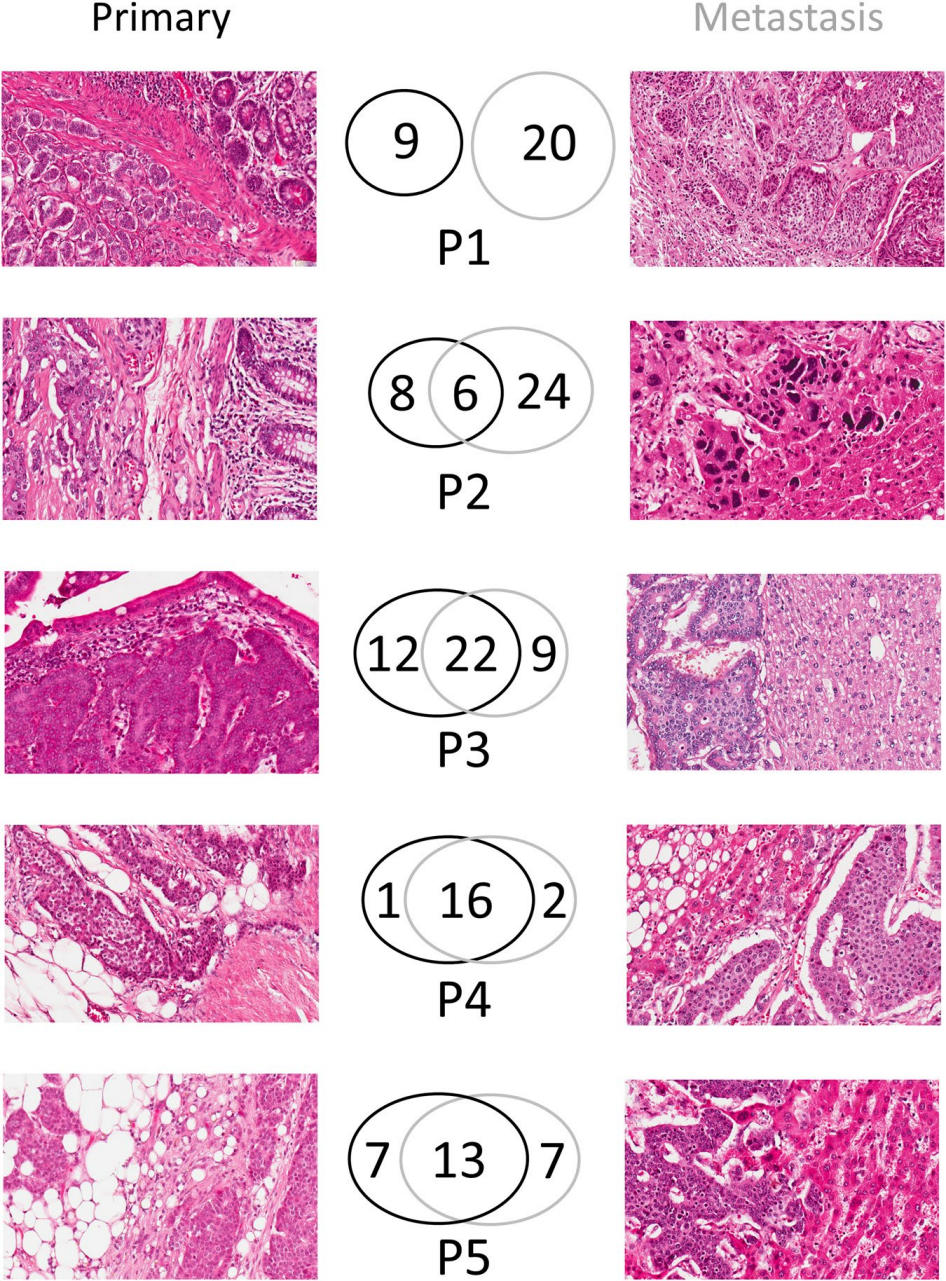
Mutational heterogeneity and representative hematoxylin and eosin stained sections of primary and metastasis. Representative areas of hematoxylin and eosin-stained slides of primary lesion (left) and metastasis (right) of small intestine neuroendocrine tumors (SI-NET) of Patient 1–5 (P1–P5). Mutations only present in the primary tumor are surrounded of black circles while mutations only present in metastasis are within grey circles. Common mutations (present in primary and metastatic SI-NET) are within the intersection. If available, areas with infiltration into benign surrounding tissue (e.g. ileal mucosa, liver tissue) were chosen. In the metastasis sample of Patient 2, only an instantaneous section was available. Magnification: 200x.

We observed a markedly differing extent of intratumoral heterogeneity of primary tumor (p) and metastasis (m) in our cohort of SI-NETs. For example, in Patient 1 (P1) no common mutations were identified (0%). On the other hand, the amount of common mutations per sample varied among the other four patients such as 20% in P2m and 94% in P4p. Amount of private mutations only present in the primary lesion but not in the metastasis varied as well strongly in Patient 2–5 between 6% (P4p) and 57% (P2p). Likewise, private mutations only detected in metastasis but not in the primary tumor varied between 11% (P4m) and 80% (P2m). An overview of mutational distribution including representative histopathological images is provided in Fig. 2.

Besides mutational analysis, distribution of observed allele frequency (AF) was examined. Across all samples with enough variants (except the metastasis sample of patient 4 that did not harbour informative variants), we observed a markedly higher AF in metastasis samples than in the primary tumor. These differences were significant in 2/4 and a trend (p > 0.10) was observed in 1/4 tested patients (false discovery rate corrected t test; p < 0.020 for patient 1, p < 0.049 for patient 2, p < 0.104 for patient 4, p < 0.085 for patient 5, Fig. 1C).

### Potential driver mutations

To assess presence of potential driver mutations, SNVs in known cancer-associated genes or genes previously described to be mutated in SI-NET were classified as likely pathogenic or variants of unknown significance according to their SIFT score (see methods for details). Thereby, sixteen likely pathogenic SNVs and two variants of unknown significance were identified. Notably, in samples with overlapping SNVs (Patient 2–5), potential driver mutations were mainly found in both samples whereas Patient 1, who had no overlap of SNVs, harbored different likely pathogenic mutations in metastasis and primary lesion (Table 2). The only recurrent non-synonymously mutated gene in this study was *RBMS3*, which was found being mutated in two patients. No marked difference in mutation pattern was observed: The most frequent SNV was C > T/G > A transition in both primary and metastatic lesions (Fig. 1B).

**Table 2:** Potential driver genes. Identified potential driver mutations within the study cohort. Genes found to be mutated within this study present as well in the cancer gene census or previous studies on small intestine neuroendocrine tumors were analyzed for potential pathogenicity (see methods for details). Mutations with a SIFT score ≤0.05 as well as nonsense or frameshift mutations were categorized as likely pathogenic, whereas the rest was classified as variants of unknown significance (VUS). Pat: patient, NA: not available.

PAT	SAMPLE	POSITION	CONSEQUENCE	GENE	SIFT SCORE	AMINO ACID CHANGE	RELEVANCE
1	metastasis	17:18188818A>AT	frameshift	TOP3A	NA	p.H538Qfs*36	Likely pathogenic
1	metastasis	4:187524330C>A	stop_gained	FAT1	NA	p.E3784*	Likely pathogenic
1	primary	5:142421455G>A	splice_region	ARHGAP26	0.002	p.D429N	Likely pathogenic
1	primary	5:158223438G>A	missense	EBF1	0.001	p.T275M	Likely pathogenic
2	metastasis	3:29323183G>A	missense	RBMS3	0.012	p.R4H	Likely pathogenic
2	metastasis	1:198703492A>T	missense	PTPRC	0.000	p.M739L	Likely pathogenic
2	metastasis	15:50784957T>G	missense	USP8	0.001	p.L765R	Likely pathogenic
2	common	17:57126678T>C	missense	TRIM37	0.126	p.N464S	VUS
2	common	2:179447188T>G	missense	TTN	0.070	p.S12934R	VUS
3	common	3:29476340C>A	missense	RBMS3	0.001	p.T61N	Likely pathogenic
3	common	19:9075291G>T	missense	MUC16	0.007	p.T4052N	Likely pathogenic
3	common	4:20599988C>A	missense	SLIT2	0.023	p.T1221N	Likely pathogenic
3	common	9:134073116G>T	missense	NUP214	0.002	p.G1412V	Likely pathogenic
4	common	12:12871070C>A	stop_gained	CDKN1B	NA	p.C99*	Likely pathogenic
4	common	7:101870749G>GT	frameshift	CUX1	NA	p.P1079Sfs*16	Likely pathogenic
4	common	6:167040464 GAGA>G	inframe	RPS6KA2	NA	p.F14del	Likely pathogenic
4	common	3:37365617T>A	missense	GOLGA4	0.003	p.I747N	Likely pathogenic
5	metastasis	9:27158095C>T	stop_gained	TEK	NA	p.R107*	Likely pathogenic
5	common	9:90258313T>A	missense	DAPK1	0.056	p.L314Q	VUS

To investigate whether mutated functional clusters differ in primary lesion and metastasis, we compared mutated genes of primary SI-NET (including data of 103 primary lesions from previous studies)^10,11^ and metastatic lesions (including data of five published metastatic lesions)^11^. We thereby found different canonical pathways to be mutated: For example, cell cycle proteins were identified in the primary lesions and gene<s of regulation of adherence junction stability and disassembly in metastasis (Supplementary Table 3).

### Copy number variation analysis

**Figure 3:**
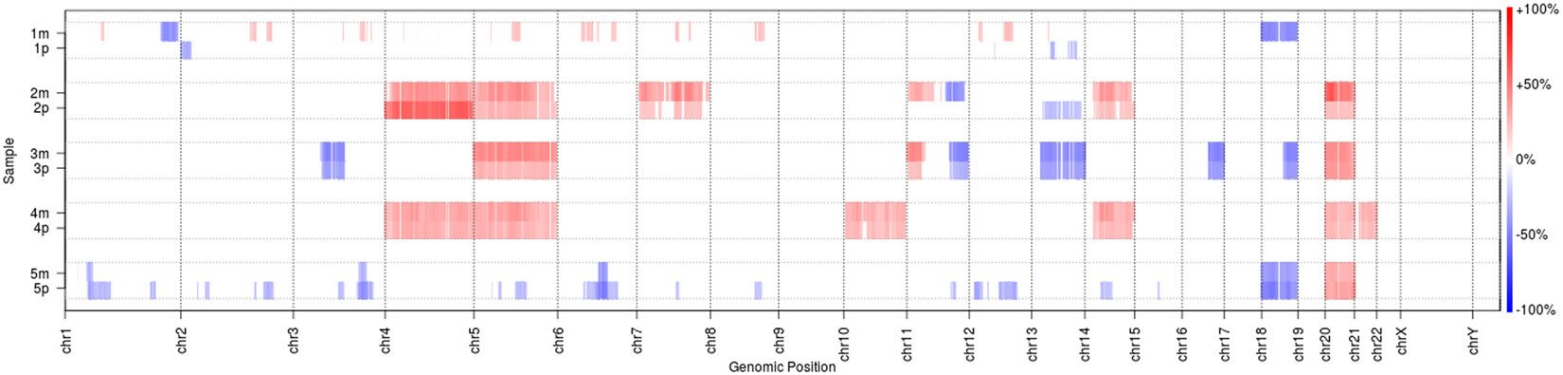
Copy number variations. Copy number variations of metastasis (m) and primary (p) of Patient 1–5. Copy number gains are shown in red, whereas losses are colored blue. Copy numbers of the control samples were subtracted and only copy number aberrations ≥20% were included (see methods for details).

The exome based copy number analysis revealed gains of chromosomes 4, 5, 7, 10, 14, 20 and 21 as well as losses of chromosome 13 and 18. Copy numbers of metastasis and primary were similar in Patient 3 and Patient 4, differed partially in Patient 2 and 5 and were completely different in Patient 1 (Fig. 3). Thereby, overlap of copy number variations (CNV) matched the overlap of SNVs in most patients (Patient 1, 2, 4, 5) but differed in Patient 3, where similar CNV alterations were observed despite heterogeneity in SNV analysis.

### Methylation analysis

**Figure 4:**
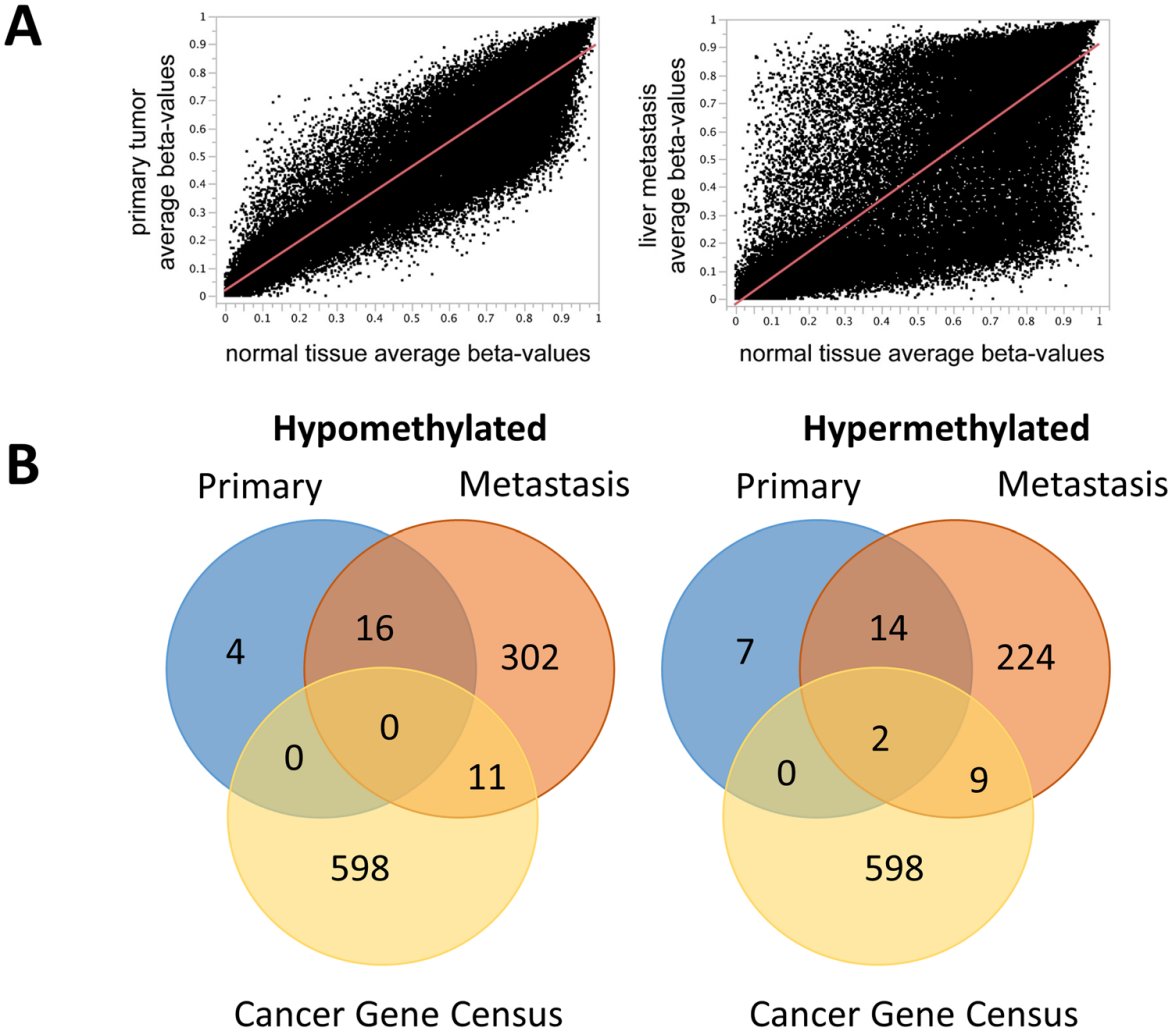
Methylation analsis of patient 1. (**A**) Average beta values of primary (left) and metastasis (right) in comparison to normal tissue of patient 1. 0 = fully unmethylated, 1 = fully methylated. (**B**) Comparison of genes harbouring promotor associated CpG sites with marked (>0.3 delta beta value) hypo- and hypermethylation in patient 1 and the COSMIC cancer gene census.

To further investigate potential background of the differing mutational profiles of primary lesion and metastasis of Patient 1, additional analysis of whole DNA methylation status of both lesions was performed and compared to benign small intestine. Whole DNA-methylome data of average beta-values revealed markedly more epigenetic dysregulation in the metastasis compared to the primary lesion (Fig. 4A). Of interest, the majority of dysregulated CpG sites of the primary were found in the metastatic lesion as well: In Fig. 4B, overlap of all genes with differentially methylated, promotor associated CpG sites is shown. Within this overlap, one hypermethylated tumor suppressor gene was identified (*KLF6*). All differentially methylated genes are shown in Supplementary Table 4.

To investigate whether these alterations might be common within SI-NETs in terms of an entity specific signature, a comparison with a control cohort of methylation data of SI-NETs (n = 20) and benign small intestine (n = 20) was performed^12^. Thereby, the common methylation alterations of Patient 1 were not found in the control cohort (Supplementary Table 5).

## Discussion

Characterization of the genetic landscape of SI-NETs is of high importance to identify potential targets for personalized therapy regimens. In the present study, primary and metastasis samples of five SI-NETs were investigated with whole exome sequencing to examine presence and extent of subclonality within this rare entity. Thereby, a profound genetic heterogeneity between primary lesions and hepatic metastasis was revealed.

A comparable mean somatic mutation rate of 0.33/Mb for primary and 0.41/Mb for metastatic lesions was observed which is within the range of previous large scale sequencing studies on SI-NET of Banck *et al*. and Francis *et al*. where a mean of 0.1/Mb and 0.77/Mb (non-silent) was reported, respectively^10,11^. This strengthens the comparability of our data with the mentioned studies, although DNA was extracted from formalin-fixed paraffin embedded (FFPE) tissue in the current study and not from frozen tissue as in the previous analyses.

While no relevant difference of the mutational pattern was observed between metastasis and primary lesion, mutational landscapes within our cohort differed clearly: A markedly varying amount of common mutations (present in both samples) as well as private mutations (not present in the corresponding sample) was identified. Moreover, in 1/5 cases no common mutations were observed at all. These observations correspond with data of Francis *et al*., where 2/3 of SI-NETs presenting with liver metastasis were found to have no common mutations in primary and metastasis^11^. In addition, AF-analysis revealed a markedly higher AF in metastasis samples than in the primary tumor, which could be explained by the metastasis growing out of a single clone from the primary while the primary has a more polyclonal population. These findings are highly remarkable since they indicate a different biological way of metastatic spread in comparison to other solid tumors. For example, recent studies on malignancies such as non-small cell lung cancer (>50% common mutations), endometrial cancer (>45%), and head and neck squamous cell carcinoma (>60%) observed high rates of common mutations in tumor and metastasis samples^14–16^. Moreover, in small cell lung cancer (SCLC), which is thought to originate from neuroendocrine cells as well, a rate of more than 95% common mutations was found suggesting a linear model of clonal evolution in this entity^14^. The observed heterogeneity has to be kept in mind for interpretation of previous genomic studies, which included only one sample of either primary or metastatic lesion. Furthermore, these data have to be considered in the planning of future biomarker-driven targeted therapy trials since results of a single biopsy might be insufficient. Copy number alterations are common events in SI-NETs. We also observed alterations in our cohort. For example, loss of chromosome 18 was found in 3/5 patients which was described to occur in >60% of SI-NETs. Likewise we found gains of chromosomes 4, 5, 7, 10, 14 and 20 as well as a loss of chromosome 13^17–20^.

In-silico pathway analysis revelead that cell cycle pathways were found to be significantly mutated in the primary lesion, whereas genes regulating adherence junction stability and disassembly were identified in the metastases group. This correlates to protein expression data of a study of Kim *et al*., where proteins involved in cell cycle and proliferation were found to be differentially expressed in primary lesions and metastasis^21^. However, functional data of metastasis development of SI-NET are still due and further studies are warranted.

A possible explanation for the presence of genetically independent primary lesion and metastasis might be common epigenetic alterations, which could potentially lead to genetic instability and consecutive development of genetic subclonality. In line with this hypothesis, a recent study profiled 49 primary SI-NET tumors with a methylation array and identified a SI-NET specific panel of epigenetically altered genes with an associated change in the expression profile of the respective gene^12^. Even so, distinct epigenetic changes were observed as well in other tumors with a high degree of common mutations such as SCLC and a definite link between epigenetic changes and the SI-NET-specific heterogeneous mutational landscape has not been determined yet^22^. To investigate a potential common epigenetic alteration of the patient without common SNVs and CNVs (P1), we performed an analysis of 850,000 methylation sites. We thereby found that the metastasis was clearly more epigenetically dysregulated in comparison to benign tissue than the primary lesion. which is in line with observations of a recent study investigating methylation profiles of SI-NET metastasis^23^. Moreover, we observed the majority of differentially (de)methylated promoter-associated CpGs of the primary to be present in the metastasis as well. Since these alterations were not found to be present in a control cohort, we hypothesize that these two lesions most likely originate from a common single clone. This suggests a common driver mutation, which is not detectable by exome sequencing. Since the majority of available sequencing data on SI-NETs is exome-based, these observations are of high interest. The only potential tumor suppressor gene being hypermethylated was *KLF6*. KLF6 depletion was associated with various cancers and a potential role in SI-NET warrants further investigation^24–27^. Unfortunately, since only FFPE tissue was available, no analysis of KLF6-expression could be performed within this study.

Besides differential methylation, other epigenetic alterations such as histone modifications or mutations in regulatory elements have to be considered as potential underlying tumor drivers warranting further investigation. In addition, since epidemiological data suggest a higher risk of SI-NET development for first-degree relatives of patients with SI-NETs, germ-line alterations might predispose for tumor development^28^. This is strengthened by the observation that SI-NETs occur multiply in up to 30%^29^. Furthermore, a familial aggregation of SI-NETs with other noncarcinoid malignancies was reported^30^. However, to date, data of genome wide association studies on potentially predisposing polymorphisms for development of SI-NETs are lacking and further investigation is needed.

In conclusion, our data demonstrate a highly varying heterogeneity of SI-NETs. Moreover, tumor drivers of SI-NET development might not be detectable by exome sequencing and further comprehensive studies including whole genome sequencing as well as epigenetic analyses are needed.

## Methods

### Patients and sample preparation

Tissue and tumor samples as well as patient data used in this study were provided by the University Cancer Center Frankfurt (UCT). Written informed consent was obtained from all patients and the study was approved by the institutional Review Boards of the UCT and the Ethics Committee at the University Hospital Frankfurt (project-number: SGI-OW-01/2013). All methods were performed in accordance with the declaration of Helsinki. Only patients with available FFPE tissue of each the primary lesion, the metastasis and the matched normal tissue were included. The definite diagnosis of SI-NET was confirmed by two expert gastrointestinal pathologists. Percentage of tumor content was assessed based on hematoxylin and eosin stained sections. After macrodissection, DNA was extracted with the Maxwell 16 FFPE tissue LEV DNA purification kit (Promega, Madison, WI) from FFPE material according to manufacturer’s recommendations. DNA yield was quantified with Quantus Fluorometer (Promega).

### Whole exome sequencing

Sequencing libraries were prepared from tumor and non-tumor tissue with SureSelectXT Human All Exon V6 (target size 60 Mb, Agilent, Santa Clara, CA) according to the manufacturer’s instructions and paired-end sequencing was performed on a HiSeq2500 (Illumina, San Diego, CA) with 2 × 100 base pairs (bp) read length. Input DNA was at least 1000 ng and at least 16 gigabases (range 16–20 Gb) of raw read data per sample were produced.

### Variant calling

Demultiplexing was performed with Illumina CASAVA (1.8.2) and adapters were trimmed with Skewer (0.1.116). Trimmed raw reads were aligned to the human genome (hg19) with the Burrows-Wheeler Aligner (BWA-mem version 0.7.2). Reads that aligned at more than one locus were discarded. Duplicate reads were removed with SAMtools (0.1.18). To enhance sensitivity, two different software tools were used for variant calling: SAMtools and varscan (2.3.5). Somatic variants in primary tumor and metastasis were selected by filtering against the variants present in the control sample. Then, all variants with a dbSNP ID were removed and only variants affecting coding exons were further evaluated. All called variants were manually reviewed in the Integrative Genomics Viewer (2.3) and all variants suspected of being technical artifacts were discarded. Only variants with an AF of ≥5% and at least ten reads were considered as true. For each mutation found only in one tumor sample, sequencing data of both the corresponding tumor and non-tumor sample were investigated for presence of reads with the same information. Alterations occurring only in either primary or metastasis were defined as private, whereas mutations present in both tumor sites were regarded as common. For analysis of AF, all variants with at least five reads were included.

### Sanger sequencing

A representative selection of SNV and insertions and deletions (Indels) covering samples of all patients and different allele frequencies was validated with Sanger sequencing. Primers were designed using NCBI Primer-Blast (http://www.ncbi.nlm.nih.gov/tools/primer-blast). PCR reaction was performed with *Taq* PCR Master Mix Kit (Qiagen, Hilden, Germany) according to manufacturer’s recommendations using 20 pmol primer and 25–50 ng template DNA. PCR reaction conditions were initial denaturation at 95 °C for 300 s, 44 cycles of 95 °C for 45 s, 56–61 °C for 60 s and 72 °C for 45 s, followed by 5 min final extension at 72 °C. The annealing temperature was chosen to be suitable for the respective primer pairs. PCR amplification was always performed for central and peripheral tumor as well as the non-tumor sample. PCR solutions were sent to Eurofins Genomics GmbH (Ebersberg, Germany) for sequencing. Primer sequences are listed in Supplementary Table 1. Validation PCR always included both primary and metastatic DNA as well as DNA of the control sample.

### Pyrosequencing

For SNVs with low allele frequencies or in case primer construction for Sanger sequencing failed, pyrosequencing of both tumor samples and corresponding non-tumor sample was performed. Primer design was performed with PSQ Assay Design (Biotage, Uppsala, Sweden) and assays were established with Pyromark Q24 (Qiagen). PCR reaction was performed with the PyroMark PCR kit (Qiagen) according to manufacturer’s recommendations using 20 pmol primer and 25–50 ng template DNA. The PCRs were performed as described for Sanger sequencing. The resulting PCR products were sequenced with the PyroMark Q24 pyrosequencer using PyroMark Gold Q96 reagents (Qiagen). All assays were run with tumor and non-tumor samples as well as positive (Qiagen human control DNA) and negative control. SNVs with ≥5% difference in mutant AF compared to non-tumor tissue and positive control were assessed as true variants.

### Identification of potential driver genes

All non-synonymous mutations detected in the current study which were present in the COSMIC cancer gene census (data extracted January 2017) were categorized as potential driver genes. Moreover, mutated genes were compared to former large scale sequencing studies on SI-NET, and recurrent genes were manually reviewed as well for potential oncogenic function^10,11^. Besides matching all non-synonymous mutations with the established list, all mutated genes were manually reviewed and those with high probability to have an oncogenic effect were examined in more detail. Mutations having passed this criteria with a SIFT score of ≤0.05, nonsense or frameshift mutations were classified as likely pathogenic mutations^31^. All other non-synonymous mutations of this list were classified as variants of unknown significance.

### Copy Number analysis

CNV were computed on uniquely mapping, non-duplicate, high quality reads using an internally-developed method based on sequencing coverage depth. Briefly, we used at least 10 reference samples to create a model of the expected coverage that represents biases introduced by the target enrichment method, sequence GC content, library preparation protocol, insert size and sequencing technology, as well as inter-sample variation.

CNV calling for germline samples was performed by computing the sample’s coverage profile, correcting for total read count and computing the deviation from the expected coverage. Genomic regions were called as variant if they deviated by at least 2 standard deviations from the model mean and the deviation was concordant with a biologically possible copy number (e.g., +50% for a heterozygous duplication, −50% for a heterozygous deletion). For tumor samples, the estimated tumor content was taken into account to deduct the copy number. For instance, given a tumor content of 60%, an observed deviation of +30% represented a heterozygous duplication in the tumor, while an observed deviation of +20% could either represent a heterozygous duplication of non-tumor tissue or a subclonal duplication in the tumor. To improve visual clarity and highlight large-scale changes, data was smoothed using the median over windows of five mega bases.

### Signal transduction pathway analysis

Functional cluster analysis was performed with the Genomatix Genome Analyzer (v3.70808, Genomatix GmbH, Munich, Germany) using the tool GeneRanker, which is based on FuncAssociate^32^. For pathway analysis, gene associations with over 400 canonical signal transduction pathways were performed collected from the Pathway Interaction Database and pathway commons^33,34^. All canonical pathways are derived from Homo sapiens. Genes were grouped into non-synonymously mutated genes in primary lesions as well as in metastasis of SI-NETs.

### Methylation analysis

Representative areas of tumor tissue were punched out of paraffin blocks. DNA was extracted using the Invisorb Genomic DNA Kit (Stratek, Birkenfeld, Germany). Amount of DNA was measured using Invitrogen’s dsDNA BR Assay Kit and Qubit System (Invitrogen, Waltham, MA). Bi-sulfite treatment was performed using EZ DNA methylation Kit from Zymo Research (Zymo Research, Irvine, CA). DNA restoration was performed using DNA Clean and Concentrator-5 (Zymo Research) and Infinium HD FFPE Restore Kit (Illumina). All following steps were performed according to Infinium HD FFPE Methylation Assay manual protocol (Illumina). Pretreated DNA was processed and hybridized on an EPIC 850k chip (Illumina). Chips were scanned on an Iscan (Illumina). Data was processed using Illumina Genome Studio as well as JMP 11 (SAS). CpG-sites with potential SNPs were removed before analyses. First we compared average beta-values, ranging between 0 (fully unmethylated) and 1 (fully methylated) of primary tumor tissue, normal tissue, and a corresponding liver metastasis. Delta beta-values of whole methylome and promoter associated regions (as annotated by the manufacturer including transcriptional start sites such as TSS200, TSS1500) were compared for primary tumor vs. normal tissue and liver metastasis vs. normal tissue.

Methylation data were compared to a control cohort of already published methylation data of SI-NET primary lesions (n = 20) as well benign small intestine (n = 20)^12^.

### Immunohistochemistry

Formalin-fixed, paraffin-embedded tissue blocks were cut into 2 µm sections and transferred to glass slides (X-tra® adhesive, Leica Biosystems, Nussloch, Germany). After drying overnight at 37 °C, slides were deparaffinized with xylene and rehydrated with ethanol. For antigen retrieval slides were either subjected to water bath for 30 min at 92 °C according to manufacturer’s instructions (Trilogy-solution 1:100; Cell Marque Corporation, Rocklin, USA) followed by washing with water and TBS buffer. Following a 3 min wash with water, the slides were then processed on the Autostainer Link 48 (Dako, Glostrup, Denmark) using an automated staining protocol (Dako EnVision™ Flex, Code K8000). All primary antibodies were monoclonal mouse antibodies (Ki-67: clone MIB-1, 1:200, Dako; Chromogranin A: clone DAK-A3, 1:800, Dako; Synaptophysin: clone DAK-SYNAP, ready-to-use; CD56: Clone 123C3, ready-to-use) and exposition time was 30 minutes. Counterstaining was performed with hematoxylin.

### Statistics

Descriptive statistics were calculated using BiAS (version 11.01, BiAS for Windows; Epsilon-Verlag, Frankfurt, Germany) and R^35^. P-values less than 0.05 were considered significant.

### Data availability

Exome data was submitted to the Sequence Read Archive (SRA) of the National Center for Biotechnology Information (NCBI) with the accession number SRP126752. All other data are available on request from the corresponding author.

## Electronic supplementary material

Supplementary Information

## References

[1] Pape U-F (2012). ENETS Consensus Guidelines for the management of patients with neuroendocrine neoplasms from the jejuno-ileum and the appendix including goblet cell carcinomas. Neuroendocrinology.

[2] Modlin IM, Lye KD, Kidd M (2003). A 5-decade analysis of 13,715 carcinoid tumors. Cancer.

[3] Yao JC (2008). One hundred years after “carcinoid”: epidemiology of and prognostic factors for neuroendocrine tumors in 35,825 cases in the United States. J. Clin. Oncol..

[4] Rinke A (2009). Placebo-Controlled, Double-Blind, Prospective, Randomized Study on the Effect of Octreotide LAR in the Control of Tumor Growth in Patients With Metastatic Neuroendocrine Midgut Tumors: A Report From the PROMID Study Group. J. Clin. Oncol..

[5] Caplin ME (2014). Lanreotide in Metastatic Enteropancreatic Neuroendocrine Tumors. N. Engl. J. Med..

[6] Yao JC (2016). Everolimus for the treatment of advanced, non-functional neuroendocrine tumours of the lung or gastrointestinal tract (RADIANT-4): a randomised, placebo-controlled, phase 3 study. Lancet.

[7] Kulke MH (2011). Future Directions in the Treatment of Neuroendocrine Tumors: Consensus Report of the National Cancer Institute Neuroendocrine Tumor Clinical Trials Planning Meeting. J. Clin. Oncol..

[8] Strosberg J (2012). Neuroendocrine tumours of the small intestine. Best Pract. Res. Clin. Gastroenterol..

[9] Strosberg J (2017). Phase 3 Trial of ^177^ Lu-Dotatate for Midgut Neuroendocrine Tumors. N. Engl. J. Med..

[10] Banck MS (2013). The genomic landscape of small intestine neuroendocrine tumors. J. Clin. Invest..

[11] Francis JM (2013). Somatic mutation of CDKN1B in small intestine neuroendocrine tumors. Nat. Genet..

[12] Karpathakis A (2016). Prognostic Impact of Novel Molecular Subtypes of Small Intestinal Neuroendocrine Tumor. Clin. Cancer Res..

[13] Alexandrov LB (2013). Signatures of mutational processes in human cancer. Nature.

[14] Saber, A. *et al*. Mutation patterns in small cell and non-small cell lung cancer patients suggest a different level of heterogeneity between primary and metastatic tumors. *Carcinogenesis* bgw128, 10.1093/carcin/bgw128 (2016). 10.1093/carcin/bgw128 27993895

[15] Gibson WJ (2016). The genomic landscape and evolution of endometrial carcinoma progression and abdominopelvic metastasis. Nat. Genet..

[16] Hedberg ML (2015). Genetic landscape of metastatic and recurrent head and neck squamous cell carcinoma. J. Clin. Invest..

[17] Kulke MH (2008). High-resolution analysis of genetic alterations in small bowel carcinoid tumors reveals areas of recurrent amplification and loss. Genes. Chromosomes Cancer.

[18] Tönnies H (2001). Analysis of sporadic neuroendocrine tumours of the enteropancreatic system by comparative genomic hybridisation. Gut.

[19] Kim DH (2008). Allelic alterations in well-differentiated neuroendocrine tumors (carcinoid tumors) identified by genome-wide single nucleotide polymorphism analysis and comparison with pancreatic endocrine tumors. Genes. Chromosomes Cancer.

[20] Andersson E, Swärd C, Stenman G, Ahlman H, Nilsson O (2009). High-resolution genomic profiling reveals gain of chromosome 14 as a predictor of poor outcome in ileal carcinoids. Endocr. Relat. Cancer.

[21] Kim MK (2016). Differential Protein Expression in Small Intestinal Neuroendocrine Tumors and Liver Metastases. Pancreas.

[22] Karlsson A (2014). Genome-wide DNA methylation analysis of lung carcinoma reveals one neuroendocrine and four adenocarcinoma epitypes associated with patient outcome. Clin. Cancer Res..

[23] Karpathakis A (2017). Progressive epigenetic dysregulation in neuroendocrine tumour liver metastases. Endocr. Relat. Cancer.

[24] Masilamani AP (2017). KLF6 depletion promotes NF-κB signaling in glioblastoma. Oncogene.

[25] Gao Y (2017). KLF6 Suppresses Metastasis of Clear Cell Renal Cell Carcinoma via Transcriptional Repression of E2F1. Cancer Res..

[26] Lièvre A (2005). Absence of mutation in the putative tumor-suppressor gene KLF6 in colorectal cancers. Oncogene.

[27] Liu X (2012). KLF6 loss of function in human prostate cancer progression is implicated in resistance to androgen deprivation. Am. J. Pathol..

[28] Kharazmi E, Pukkala E, Sundquist K, Hemminki K (2013). Familial Risk of Small Intestinal Carcinoid and Adenocarcinoma. Clin. Gastroenterol. Hepatol..

[29] Capella, C, Arnold, R, Klimstra, D. In *WHO classification of tumors of the digestive system*. (ed. Bosman, F. T., Carneiro, F., Hruban, R. H., Theise, N.) 102–107 (IARC, 2010). at, http://apps.who.int/bookorders/anglais/detart1.jsp?codlan=1&codcol=70&codcch=4003.

[30] Hiripi E, Bermejo JL, Sundquist J, Hemminki K (2009). Familial gastrointestinal carcinoid tumours and associated cancers. Ann. Oncol..

[31] Kumar P, Henikoff S, Ng PC (2009). Predicting the effects of coding non-synonymous variants on protein function using the SIFT algorithm. Nat. Protoc..

[32] Berriz GF, King OD, Bryant B, Sander C, Roth FP (2003). Characterizing gene sets with FuncAssociate. Bioinformatics.

[33] Schaefer CF (2009). PID: the Pathway Interaction Database. Nucleic Acids Res..

[34] Cerami EG (2011). Pathway Commons, a web resource for biological pathway data. Nucleic Acids Res..

[35] R Core Team. *R: A Language and Environment for Statistical Computing*, http://www.r-project.org/ (2016).

